# Alexandr Pavlovich Rasnitsyn, (palaeo)entomologist extraordinaire – a personal appreciation

**DOI:** 10.3897/zookeys.130.1890

**Published:** 2011-09-24

**Authors:** Denis J. Brothers

**Affiliations:** School of Biological and Conservation Sciences, University of KwaZulu-Natal, Pietermaritzburg, Private Bag X01, Scottsville, 3209 South Africa

This note is not meant to be an exhaustive account of the many achievements of Alex Rasnitsyn over the 75 years of his life thus far. Apart from anything else, I have little knowledge of his activities outside of my professional interactions with him. Also, there will undoubtedly be several other biographical essays this year to celebrate his accomplishments in many fields. So, my intention, after providing the essential details to place him in context, mainly derived from his personal page on the Paleontological Institute’s website (Rasnitsyn 2011) and a note in the Paleontological Journal (Laboratory of Arthropods 2011), is to give my personal impressions of our interactions, his influence on my own life and work, and on hymenopterology in general.

**Figures 1–5.**
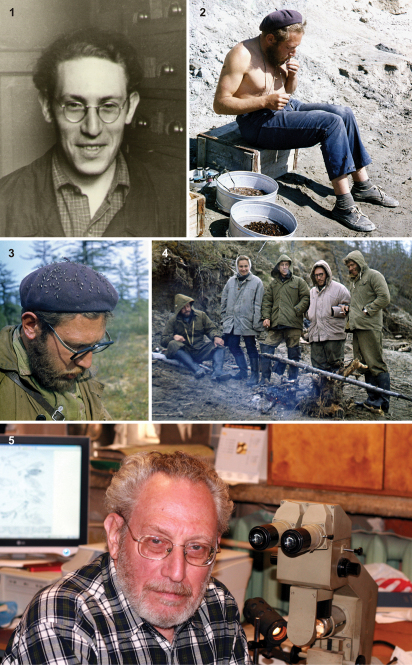
Alex Rasnitsyn in Russia, in the laboratories of the Paleontological Institute, Moscow, and in the field. **1** In 1961 (photo by Oleg Amitrov). **2** In 1971, sorting amber at Yantardakh, Maimecha River, Taymyr Peninsula, northern Siberia (71°18'30"N 99°34'12"E) (photo by Alexandr Ponomarenko). **3** In 1971, with mosquitoes near Yantardakh (photo by Alexandr Ponomarenko). **4** In 1971, at Gubina Gora near Khatanga, Taymyr Peninsula (71°59'54"N 102°34'22"E); left to right: Alexandr Ponomarenko, Irina Sukacheva, Pol' Perov, Alex Rasnitsyn, Vladimir Zherikhin (photo probably by Anatoly Mikheev). **5** In 2008 (photo by Roman Rakitov).

Alexandr Pavlovich Rasnitsyn was born in Moscow, Russia, on 24 September, 1936, and has lived there since. His interest in insects and general natural history soon became apparent, and he joined the Club of Young Biologists at the Moscow Zoo. In 1955 he enrolled at the Moscow State University, and in 1960 graduated with honours and a masters degree in entomology, his thesis being on “Hibernation in the ichneumon flies subfamily Ichneumoninae”, showing that his passion for Hymenoptera was developed right from the start. That same year he joined the Arthropoda Laboratory, headed by Professor Boris Rohdendorf, in the Paleontological Institute of the USSR (now Russian) Academy of Sciences, Moscow. From this start as a young man (Figure 1), he worked his way up sequentially from positions as Technician, Junior and Senior Research Worker, to becoming the Head of the Laboratory (1979–1996), Principal Research Worker (since 1996) and then again Head of the Laboratory (from 2002 after the sudden death of his successor, Vladimir Zherikhin, to the present, Figure 5). He has thus headed the most productive and influential group of palaeoentomologists in the world for 28 years. During his employment he also earned two doctorates from the Paleontological Institute, a PhD in 1967 on “Mesozoic Hymenoptera Symphyta and early evolution of Xyelidae”, and a DSc in 1978 on “Origin and evolution of Hymenoptera”. In 1991 he was awarded the title of Professor, and in 2001 he was given the award of “Honoured Scientist” by the Russian Federation. He served as the first President of the International Palaeoentomological Society (2001–2005), and was given Honorary Membership of the Russian Entomological Society (2004) and has been a member of their Council since 2007. He has long held an honorary appointment at the Natural History Museum, London, England.

By his own account, Alex’s interests encompass not only the palaeontology, phylogeny and taxonomy of Hymenoptera and of insects in general, but also broader biological problems, including evolutionary theory, dynamics of taxonomic diversity, and methodology of phylogenetics, taxonomy, and nomenclature. His fascination with the natural world is unquenchable, as shown by his participation in or leading collecting expeditions over 22 field seasons (starting in 1956) to many famous fossil-insect localities in Central Asia, North Caucasus, Siberia (Figures 2–4), Transbaikalia, Mongolia, England, Germany, USA and Israel. He has also visited collections to study specimens, both fossil and modern, in many countries: Canada, China, Denmark, England, France, Germany, Poland, South Africa, Spain and USA. He has participated in many conferences around the world and in several international research collaborations.

Alex’s incredible productivity and breadth of interests are graphically shown by his publications. The most complete list available to me, a late precursor to Engel and Shcherbakov (2011), includes 366 items produced from 1959 onwards. This output is both prodigious and diverse. I broke the items down into ten broad categories and discovered the following, arranged in descending order of items per category (Table 1):

**Table 1.** Items authored or co-authored by Alex Rasnitsyn, including abstracts.

Field of contribution	Year of first item	Year of latest item	Number of items	Items per year	Median year	Modal year
Palaeontology of Hymenoptera	1963	2011	117	2.39	2000	2000
Palaeontology of other insects	1974	2011	111	2.92	2002	2002
Theory of systematics	1966	2010	36	0.80	1992	1991
Theory of evolution	1965	2005	20	0.49	1974	1971
Modern Hymenoptera	1959	2010	17	0.33	1986	1981
Evolution of Hymenoptera	1965	2006	15	0.36	1980	1971
Ecology, biodiversity, etc.	1963	1995	15	0.45	1989	1989
Miscellaneous topics	1977	2008	13	0.41	2003	2004
Evolution of insects	1976	2003	11	0.39	1996	1976
Editorial work (books)	1980	2008	11	0.38	1988	1985
Total	1959	2011	366	6.91	1997	2002

Obviously, some items would span more than one category, but I have merely assigned each to what I judged to be the single most relevant category. Of the 366, 189 have Alex as sole author, 105 have two authors, 43 have three, 14 have four, 12 have five to nine, and 3 (tributes or obituaries) have more than twenty authors; the average number is 2.1. The frequency of co-authorships has increased over the years, from an average of 1.3 over the first 20 years to 2.6 for the last 20, indicating increasing requests for collaboration from colleagues. The above items include co-authorship with 187 different colleagues, a remarkable spread.

It is no surprise that most of Alex’s contributions have been on Hymenoptera, specially their palaeontology, but also on modern groups and, probably of most general influence, the evolution of the order, the relatively few items in that category building on the others and comprising large and major contributions (specially Rasnitsyn 1969, 1980, 1988). He has produced 149 items on Hymenoptera, at a rate of 2.81 per year since 1959, the median and modal years being 1999 and 2000 respectively. Not only his sheer productivity, but even more his encyclopaedic coverage and integration of his ideas into pioneering systems outlining the evolution of the members of the order, mean that he is unquestionably the greatest current authority on the Hymenoptera as a whole. His analyses, relying extensively on fossils, often produced results somewhat different from those obtained by cladistic methods using the morphology of modern representatives, including some of my own (e.g. Brothers 1999); even cladistic re-analyses using his own characters produced less conclusive results (Ronquist et al. 1999), reflecting the difficulty of integrating information about incomplete fossils and modern forms in a single analysis. Nevertheless, many of his proposed groupings are corroborated in some of the latest analyses incorporating both morphological and molecular information from representatives across the order (e.g. Sharkey et al. 2011), illustrating his remarkable insights and unique encyclopaedic knowledge of the order as a whole. Alex’s unparalleled breadth and integration of research on the Hymenoptera was formally recognised in 2008 by the award of the Distinguished Research Medal of the International Society of Hymenopterists, only the fourth such award, following similar recognition of the legendary Charles Michener, Zdeněk Bouček and Richard Bohart.

I was surprised, however, to discover that Alex’s contributions to the palaeontology of other insect groups, although starting about 15 years after his first contribution on Hymenoptera, have been almost as numerous as those on hymenopteran palaeontology, with the same average number of co-authors (one), and at a higher annual rate over the period of their production. The groups covered range across the entire spectrum of insect orders, including several enigmatic ones whose relationships remain obscure. In all of the other categories his output has been much less and generally earlier in his career, although he has continued to make contributions in all (except for more ecological areas) until recently. Although the bulk of his editorial work was done before about 1991, it included two very significant and influential compilations on insect palaeontology and evolution (Rohdendorf and Rasnitsyn 1980, and Rasnitsyn and Quicke 2002), the latter being the first large-scale work of its sort in English. He has developed and propounded a mode of systematic analysis to estimate the pathways of evolution and phylogenies which is considered idiosyncratic by many, but which is firmly grounded in his extensive knowledge of fossil insects, and which certainly provides an important alternative way of looking at such issues, a recent paper (Rasnitsyn 2010) providing comparisons between various methods. The accompanying list of Alex’s publications (Engel and Shcherbakov 2011) must be consulted for a proper appreciation of the volume and variety of his contributions.

It is obvious from the above that Alex has a fearsome intellect and exhibits boundless dedication and hard work in pursuit of his passion. He is much sought as a collaborator, and he is certainly not slowing down – he produced 19 items in 2010 alone. One might consider that such a person must be self-centred, forceful and entirely focused on his work, but this is not true of Alex. He is a real gentleman, very thoughtful of the needs of others, humble, and ready to participate in non-work-related activities which might expand his appreciation and understanding of the natural world. These are qualities which I have experienced in my many interactions with him.

I first met Alex in 1988 at the XVIII International Congress of Entomology and meeting of the International Society of Hymenopterists, in Vancouver, Canada. We both enjoyed the exchanges with colleagues and chatted about our mutual interests in hymenopteran phylogeny (mine restricted to Aculeata) but prospects for closer collaboration seemed poor, given the political systems in place at the time. At that stage I had no idea that I might become involved in hymenopteran palaeontology myself. The turning point for me came in early 1991 when I was made aware of an extensive collection of Cretaceous insect fossils from the Orapa diamond mine in Botswana, which were housed under the care of Dr Richard Rayner, a palaeobotanist, at the Bernard Price Institute of Palaeontology (BPI), University of the Witwatersrand, Johannesburg, South Africa. This piqued my curiosity, and I was able to look at several of the blocks containing the most spectacular of the insect fossils. I was immediately struck by a most beautifully preserved specimen of some sort of wasp, which I was able to photograph. On my return home, I fortuitously found the latest issue of the journal Psyche on my desk, and on looking through it discovered that the first paper dealt with Mesozoic Vespidae (Carpenter and Rasnitsyn 1990). I immediately compared my photographs with the figures in the paper, and became convinced that the Orapa specimen was a new species of *Curiosivespa*, a genus previously known only from north-eastern Asia. This was an exciting serendipitous discovery and led to my first palaeoentomological paper (Brothers 1992).

As a consequence, I had even more in common with Alex when we met again later in 1991 at the Second Quadrennial Meeting of the International Society of Hymenopterists in Sheffield, England. We were also both at the Third Quadrennial Meeting of the Society in Davis California, USA in 1995. Then, in 1998, we were both participants in an important symposium and workshop investigating the phylogeny of Hymenoptera, organised by Fredrik Ronquist in Uppsala, Sweden (see Ronquist 1999), part of it devoted to reanalysing Alex’s ideas from a rigorous cladistic perspective, with somewhat different results from his original proposals (see Ronquist et al. 1999). A lesser person might have taken this personally, as some sort of repudiation of their work, but Alex, as the true scientist that he is, considered it to provide new perspectives which warranted further exploration, and, perhaps in response, soon produced a paper analysing ways to test cladograms using fossils (Rasnitsyn 2000). Also in 1998 the First Palaeoentomological Conference was held, in Moscow at the home of the world’s greatest concentration of palaeoentomologists. It was an eye-opener for someone like me who had entered the field late and almost accidentally, not least because of Alex’s evident mastery of the entire field and his varied contributions. A lasting memory of that visit is Alex’s and his wife, Irina Goncharova’s, hospitality. I had stayed on for a few days after the conference and was invited, together with Conrad Labandeira, to share a Sunday with them. This involved a walk from their apartment across the multi-lane road encircling the city, past several rather run-down farmhouses and into the forest, Alex carrying a heavy load of equipment and food items. After walking for a considerable distance, collecting wild mushrooms and investigating unfamiliar biological phenomena along the way, we reached a small clearing where Alex collected firewood and made a fire for our lunch. We enjoyed a marvellous relaxing time, eating and drinking the excellent provisions and chatting about many topics, before we had to make our way back. We then spent more time in their apartment, being provided with further refreshments. Thanks again, Alex and Irina, for that wonderful afternoon.

In 1999 I was sent an Eocene fossil wasp wing from Canada for my opinion on its placement, and I immediately referred it to Alex, who identified it as a new genus of primitive sphecid and suggested that it be included in a paper he was currently involved with; Pulawski et al. (2000) was the result. At about the same time Alex told me about a couple of specimens from New Jersey Cretaceous amber which he thought were Plumariidae (putatively the first fossils for that family) and arranged for them to be sent to me. I initially agreed with this placement, but after various analyses became convinced that they should probably rather be placed in a separate closely related family, described in an accompanying paper as Plumalexiidae (Brothers 2011).

After 1992 I was unable to do any further work on the Orapa material (it being housed 500 km from my home in Pietermaritzburg) for a long time. But I remained painfully aware that there was a treasure trove of Cretaceous material awaiting study in Johannesburg, and could think of no-one better to evaluate its significance than Alex. So I managed to secure funding in 2001 to invite him as a plenary speaker to an entomological conference in Pietermaritzburg and then to spend about three weeks examining the fossils in Johannesburg. During that period I scanned about 2000 rock pieces (containing about 5000 insect fossils) for Hymenoptera and Alex examined the 68 blocks on which I had found at least one hymenopteron, identifying and listing all the fossils and making drawings of the 108 hymenopterons found. I also managed to photograph them. Alex’s experience, persistence and encyclopaedic knowledge permitted us to make an estimate of the insect diversity in the collection (Brothers and Rasnitsyn 2003), and laid the basis for our further collaborative work on the Orapa fossil Hymenoptera (Dlussky et al. 2004; Rasnitsyn and Brothers 2007; Brothers and Rasnitsyn 2008; Rasnitsyn and Brothers 2009; Kopylov et al. 2010), work as yet far from completion.

The year 2001 saw further contact, since we both attended the Second International Congress on Palaeoentomology in Krakow, Poland, where Alex was elected the first President of the International Palaeoentomological Society to much acclaim at its founding meeting, and where it was decided that the next such congress would be held in South Africa. This provided another opportunity for him to visit me, and in 2005 he spent some time in Pietermaritzburg, courtesy of funding from the South African government in support of Russian academics visiting our country, before Fossils X 3: 3rd International Congress of Palaeoentomology with 2nd International Meeting on Palaeoarthropodology and 2nd World Congress on Amber and its Inclusions, held in Pretoria. He was also able to do further work on the Molteno Formation (Triassic) fossils amassed by John Anderson at the South African National Botanical Research Institute in Pretoria (a collection now also housed at BPI, Johannesburg). Then Alex came to South Africa again in 2006 for the Sixth International Conference of Hymenopterists, held at Sun City.

**Figures 6–8.**
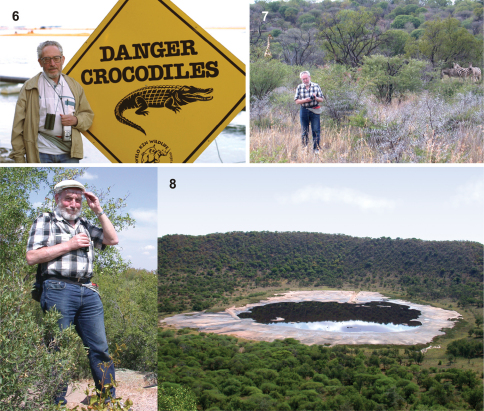
Alex Rasnitsyn in South Africa. **6** At the Lake St Lucia estuary mouth, northern KwaZulu-Natal (28°22'58"S 32°25'12"E), July 2001 (photo by Justin Waldman). **7** At Buffelsdrift residential nature reserve, north of Pretoria, Gauteng Province (25°33'41"S 28°20'48"E), February 2005 (onlookers added although also photographed there). **8** Surveying the Tswaing meteorite crater (right), north of Pretoria, Gauteng Province (25°24'38"S 28°05'14"E), February 2005.

I have thus been extremely fortunate to benefit from extensive interactions with Alex, to have him sharing his vast knowledge of Hymenoptera diversity, and patiently explaining palaeontological conventions and practices to someone without any training in palaeontology or even geology. I have always been amazed at his readiness to spend time addressing my concerns and yet obviously being able to spend even more time simultaneously on all his other projects. Nothing has been too much trouble for him. I can only assume that the level of interaction I have had has extended to all of his other collaborators, a very wide diversity of people from all over the world. Obviously, colleagues in all areas of palaeoentomology turn to his advice and participation when faced with interesting problems. To my mind, he embodies the ideal scientist, someone filled with an inexhaustible curiosity about the natural world and its history, able to focus intently on the task at hand and yet able to interrupt that task when necessary and return to it as if the interruption never happened (a skill needed by any manager), and also to enjoy doing something completely different when the opportunity arises. I remember his delight when we travelled from Pietermaritzburg to Johannesburg in 2001 and were able to visit places such as St Lucia (Figure 6) and the Hluhluwe-Mfolozi game reserve where we stayed overnight. He was thrilled by the diversity of game animals and birds, from rhinoceros, elephant and buffalo to oxpeckers and weavers. When we had to leave the reserve he leaned back and said he had been “Alex in Wonderland”. The same enjoyment was in evidence in 2005 when we stayed for a few nights at an eco-estate north of Pretoria (Figure 7) and also when we visited the impact crater about 1 km in diameter and 100 m deep at Tswaing (Figure 8), also north of Pretoria.

The celebration of Alex’s 75th birthday is a wonderful opportunity to look back at his many accomplishments, let him know how much we all appreciate them, and admire his continuing energy and drive in pursuit of his passion. Long may it continue. Happy birthday, Alex, and we certainly wish you many more to come.
